# Application of video-assisted thoracic surgery in the standard operation for thoracic tumors

**DOI:** 10.7497/j.issn.2095-3941.2013.01.005

**Published:** 2013-03

**Authors:** Ju-Wei Mu, Gui-Yu Chen, Ke-Lin Sun, Da-Wei Wang, Bai-Hua Zhang, Ning Li, Fang Lv, You-Sheng Mao, Qi Xue, Shu-Geng Gao, Jun Zhao, Da-Li Wang, Zhi-Shan Li, Wen-Dong Lei, Yu-Shun Gao, Liangze Zhang, Jin-Feng Huang, Kang Shao, Kai Su, Kun Yang, Liang Zhao, Fei-Yue Feng, Yong-Gang Wang, Jian Li, Jie He

**Affiliations:** Department of Thoracic Surgical Oncology, Cancer Institute & Hospital, Chinese Academy of Medical Sciences, Beijing 100021, China

**Keywords:** Video-assisted thoracic surgery (VATS), non-small cell lung cancer (NSCLC), esophageal cancer, thymoma

## Abstract

**Objective:**

To evaluate the short-term outcomes of video-assisted thoracic surgery (VATS) for thoracic tumors.

**Methods:**

The data of 1,790 consecutive patients were retrospectively reviewed. These patients underwent VATS pulmonary resections, VATS esophagectomies, and VATS resections of mediastinal tumors or biopsies at the Cancer Institute & Hospital, Chinese Academy of Medical Sciences between January 2009 and January 2012.

**Results:**

There were 33 patients converted to open thoracotomy (OT, 1.84%). The overall morbidity and mortality rate was 2.79% (50/1790) and 0.28% (5/1790), respectively. The overall hospitalization and chest tube duration were shorter in the VATS lobectomy group (*n*=949) than in the open thoracotomy (OT) lobectomy group (*n*=753). There were no significant differences in morbidity rate, mortality rate and operation time between the two groups. In the esophageal cancer patients, no significant difference was found in the number of nodal dissection, chest tube duration, morbidity rate, mortality rate, and hospital length of stay between the VATS esophagectomy group (*n*=81) and open esophagectomy group (*n*=81). However, the operation time was longer in the VATS esophagectomy group. In the thymoma patients, there was no significant difference in the chest tube duration, morbidity rate, mortality rate, and hospital length of stay between the VATS thymectomy group (*n*=41) and open thymectomy group (*n*=41). However, the operation time was longer in the VATS group. The median tumor size in the VATS thymectomy group was comparable with that in the OT group.

**Conclusions:**

In early-stage (I/II) non-small cell lung cancer patients who underwent lobectomies, VATS is comparable with the OT approach with similar short-term outcomes. In patients with resectable esophageal cancer, VATS esophagectomy is comparable with OT esophagectomy with similar morbidity and mortality. VATS thymectomy for Masaoka stage I and II thymoma is feasible and safe, and tumor size is not contraindicated. Longer follow-ups are needed to determine the oncologic equivalency of VATS lobectomy, esophagectomy, and thymectomy for thymoma *vs*. OT.

## Introduction

Video-assisted thoracic surgery (VATS) has become an important part of minimally invasive thoracic surgery. VATS has been proved safe and effective for a large variety of thoracic tumors. A prospective multi-institutional study^1^ and 2 meta-analyses^2^^,^^3^ of the Cancer and Leukemia Group B revealed VATS lobectomy as an appropriate procedure for early-stage non-small cell lung cancer (NSCLC) compared with open thoracotomy (OT). VATS lobectomy can be performed with low postoperative complications and mortality, and it has a similar 5-year survival rate to that of OT lobectomy, demonstrating similar oncologic efficacies. However, there are controversies on the safety and efficacy of minimally invasive esophagectomy (MIE) for esophageal cancer^4^^,^^5^ and minimal invasive thymectomy for thymoma^6^^,^^7^. In the present study, the safety and feasibility of VATS pulmonary lobectomy, VATS esophagectomy, and VATS thymectomy were explored.

## Materials and methods

### Clinical data

The medical records of 1,790 consecutive patients were reviewed. These patients underwent VATS pulmonary resections, VATS esophagectomies, VATS resections of mediastinal tumors, and VATS biopsies at the Cancer Institute & Hospital, Chinese Academy of Medical Sciences between January 2009 and January 2012. This study was approved by the Institutional Review Board of the Cancer Institute & Hospital, Chinese Academy of Medical Science. In the same period, 753 lobectomies, 81 esophagectomies, and 41 thymectomies via OTs (OT group) were randomly selected as controls. The clinical variables of paired groups were compared, including age, sex, tumor location, forced expiratory volume in one second (FEV_1_), FEV_1_%, operation time, number of lymph nodal dissection (LND), number of lymph node stations, chest tube duration, morbidity, mortality, and hospital length of stay (LOS). Lung cancer and esophageal cancer staging were carried out according to AJCC 2009 cancer staging^8^^,^^9^. Thymoma staging were carried out according to Masaoka thymoma staging^10^.

### Surgery

#### Pulmonary resection

Techniques of VATS resection for NSCLC have been described in our previous report^11^ and are outlined as follows. General anesthesia with selective lung ventilation was performed using a double lumen endotracheal tube. Patients were placed in a lateral decubitus position on the operation table. In the VATS group, one thoracoport was initially placed in the seventh or eighth intercostal space on the middle axillary line as observation port. A window (3-6 cm long) was used as the utility incision. The thoracotomy was opened centering around the third or fourth intercostal space with no rib spreading. The third port hole was made based on the location of the tumor. Finally, systematic LND was performed. In the OT group, conventional posterolateral incisions were made for lobectomy (20-30 cm long) and pulmonary wedge resection (10-20 cm long). All specimens were placed in an impermeable bag and removed through the utility incision.

### Esophagectomy

VATS esophagectomy was performed by the method of Luketich *et al*.^12^. VATS esophagectomy includes total VATS esophagectomy and hybrid VATS esophagectomy. The former consists of thoracoscopic esophagectomy, laparoscopic gastric preparation, and cervical anastomosis; the latter comprises only thoracoscope surgery. In the OT group, esophagectomy was performed by the Mckeown method^13^.

### Thymectomy

VATS thymectomy was performed by the method of Landreneau *et al*.^14^ and OT thymectomy via sternotomy, or anterior thoracotomy according to the thymoma location.

### Statistical analysis

The SPSS software package 13.0 for Windows was used for the statistical analysis. Data were presented as median values (interquartile range) for continuous variables and as percentages for dichotomous variables. Continuous variables were analyzed by the *t*-test or nonparametric test, and categorical variables were analyzed by the χ^2^ test. The significant level was set as a *P* value less than 0.05.

## Results

This cohort included 1,790 patients (1,206 males and 584 females) with a median age of 59 years (19 to 86 years). The overall morbidity rate was 2.79% (50/1,790). The complications included severe cardiac arrhythmia requiring medication (*n*=23), respiratory failure (*n*=6), postoperative bleeding (*n*=5), pneumonia (*n*=2), atelectasis (*n*=3), bronchial pleural leakage (*n*=2), bronchial leakage after esophageal surgery (*n*=1), esophageal anastomotic leakage (*n*=2), and wound infection (*n*=6). The mortality rate was 0.28% (5/1,790). Three patients with lung cancer died of acute respiratory distress syndrome, one patient with lung cancer died of pulmonary embolism, and one patient with esophageal cancer died of bronchial leakage.

VATS resections were performed in 1,790 patients, with 33 conversions to OT as a result of pleural adhesion (*n*=11) and intraoperative bleeding [bleeding of the pulmonary artery branch (*n*=13), pulmonary vein (*n*=5), azygous vein (*n*=2), and innominate vein (*n*=2)]. The VATS procedures and diagnosis of all patients are summarized in **Table 1**.

**Table 1:** Diagnosis and procedures for 1,790 patients who underwent VATS

Diagnosis	Procedure	Number
NSCLC	Lobectomy	949
	Pneumonectomy	9
	Wedge resection	306
	Bilobectomy	17
	Sleeve lobectomy	5
	segmentectomy	16
	Biopsy	8
SCLC	Lobectomy	16
	Wedge resection	6
Pulmonary metastasis	Lobectomy	12
	Wedge resection	63
Benign lung tumor, inflammatory lung disease and congenital lung disease	Lobectomy	40
	Wedge resection	104
	Biopsy	2
Esophageal cancer	Total VATS esophagectomy	42
	Hybrid VATS esophagectomy	39
Esophageal stromal tumor	Complete resection	5
Thymoma	Complete resection	41
	Biopsy	13
Thymic cancer	Complete resection	4
Thymic hyperplasia	Biopsy	7
	Complete resection	21
Germ cell tumor	Complete resection	6
	Biopsy	1
Mediastinal cyst	Complete resection	5
Lymphoma, small cell cancer and metastatic tumor	Biopsy	6
Neurogenic tumor	Complete resection	25
Pleural fibrous tumor	Complete resection	3
Metastatic pleural tumor	Biopsy	19

The overall hospitalization and chest tube durations were shorter in the VATS lobectomy group than in the OT lobectomy group, but the operation time was longer in the VATS lobectomy group. There was no significant difference in the morbidity and mortality rates between the two groups. There was no significant difference in the number of LND and number of lymph node stations between the VATS lobectomy and OT lobectomy groups for pathological stage I and II NSCLC. However, there was a significant difference in the number of lymph node stations in pathological stage III between the two groups (**Table 2**).

**Table 2:** Comparison of short-term outcomes between VATS lobectomy and open lobectomy for NSCLC (*n*=1702)

Characteristics	VATS group (*n*=949)	OT group (*n*=753)	*P*
Age (years)	59 (52-65)	57 (52-61)	<0.001
Male (%)	583 (61.4)	439 (58.3)	0.195
History of smoking (%)	368 (38.8)	315 (41.8)	0.213
Charlson scores	0 (0-1)	0 (0-0)	<0.001
Tumor location (%)			0.998
LUL	232 (24.4)	189 (25.1)	
LLL	167 (17.6)	132 (17.5)	
RUL	273 (28.8)	212 (28.2)	
RML	58 (6.1)	47 (6.2)	
RLL	219 (21.3)	173 (23.0)	
FEV_1_ (L)	2.38 (2.05-2.89)	2.60 (2.37-3.01)	<0.001
FEV_1_ (%)	70.4 (69.2-88.9)	91.7 (82.1-101.0)	<0.001
Operation time (h)	2.6 (2.0-3.3)	2.7 (2.3-3.2)	0.074
No. of LND			
Stage I	16 (11-24)	15 (10-21)	0.647
Stage II	20 (15-24)	23 (19-25)	0.055
Stage III	19.0 (10.5-30.0)	21.0 (18.0-24.0)	0.139
No. of station of LND			
Stage I	6 (4-7)	5 (4-6)	0.122
Stage II	6 (5-7)	6 (5-7)	0.679
Stage III	4 (3-4)	6 (5-6)	<0.001
Histology (%)			0.003
Squamous cell carcinoma	226 (23.8)	228 (30.3)	
Adenocarcinoma	672 (70.8)	47.5 (63.1)	
Others	51 (5.4)	50 (6.6)	
Chest tube duration (d)	4.0 (4.0-5.5)	5.0 (4.0-6.0)	<0.001
Pathological stage (%)			0.520
I	683 (72.0)	493 (65.5)	
II	141 (14.9)	140 (18.6)	
III	125 (13.2)	120 (15.9)	
Morbidity (%)	23 (2.4)	25 (3.3)	0.303
Mortality (%)	4 (0.4)	2 (0.3)	0.699
Hospital LOS (d)	15.0 (13.5-18.0)	17.0 (15.0-20.0)	<0.001
Postoperative LOS (d)	9.0 (8.0-11.0)	11.0 (9.0-12.0)	0.205

VATS, video-assisted thoracic surgery; OT, open thoracotomy; LUL, left upper lobe; LLL, left lower lobe; RUL, right upper lobe; RML, right middle lobe; RLL, right lower lobe; FEV1, forced expiratory volume in one second; LND, lymph node dissection; LOS, length of stay.

In the VATS esophagectomy group, there were 42 esophagectomies and 39 hybrid esophagectomies. No significant difference was found in the number of nodal dissection, chest tube duration, morbidity rate, mortality rate, or hospital LOS between the VATS esophagectomy and open esophagectomy (OE) groups. Patients who underwent VATS esophagectomies had longer operation time (**Table 3**).

**Table 3:** Comparison between the short-term outcomes of VATS esophagectomy and OE for esophageal cancer (*n*=162)

Characteristics	VATS group (*n*=81)	OE group (*n*=81)	*P*
Age (years)	60.0 (54.0-66.0)	56.0 (52.0-60.5)	0.004
Male (%)	49 (60.5)	53 (65.4)	0.626
Charlson scores	0 (0-0.0)	0 (0-0.0)	0.638
Tumor location (%)			0.620
Upper	33 (40.7)	30 (37.0)	
Mid	40 (49.4)	39 (48.1)	
Lower	8 (9.9)	12 (14.8)	
Operation time (h)	6.7 (5.0-8.0)	5.0 (3.0-5.0)	<0.001
Number of nodal dissection	15 (9-23)	16 (13-18)	0.172
Chest tube duration (d)	5 (3-7)	4 (3-7)	0.154
Pathological stage (%)			0.931
I	25 (30.9)	23 (28.4)	
II	33 (40.7)	35 (43.2)	
III	23 (28.4)	23 (28.4)	
Morbidity (%)	12 (14.8)	11 (13.6)	1.000
Mortality (%)	1 (1.2)	1 (1.2)	1.000
Hospital LOS (d)	18 (15-21)	21 (16-21)	0.833

VATS, video-assisted thoracic surgery; OE, open esophagectomy; LOS, length of stay.

There was no significant difference in the chest tube duration, morbidity rate, mortality rate, and hospital LOS between VATS thymectomy and open thymectomy for thymoma. Patients who underwent VATS thymectomies had longer operation time than those who underwent OT thymectomy. The median tumor size of 5.4 cm in the VATS group was comparable with that of 4.5 cm in the open thymectomy group (**Table 4**).

**Table 4:** Comparison between the short-term outcomes of VATS thymectomy and open thymectomy for thymoma (*n*=82)

Characteristics	VATS thymectomy group (*n*=41)	OT thymectomy group (*n*=41)	*P*
Age (years)	52 (41-61)	52 (40-57)	0.419
Male (%)	23 (56.1)	16 (39.0)	0.184
Charlson scores	0 (0-1)	0 (0-0)	0.007
Operation time (h)	2.1 (1.3-3.0)	2.0 (1.7-2.0)	0.038
Chest tube duration (d)	3 (3-4)	3 (3-4)	0.343
Tumor size (cm)	5.4 (4.0-7.5)	5 (3.5-6.0)	0.081
Myasthesia gravis	4 (9.8)	5 (12.2)	1.000
Masaoka staging (%)			0.269
Stage I	17 (41.5)	23 (56.1)	
Stage II	24 (58.5)	18 (43.9)	
Morbidity (%)	0 (0)	4 (9.8)	0.116
Mortality (%)	0 (0)	0 (0)	1.000
Hospital LOS (d)	12 (9-15)	12 (9-15)	0.650

VATS, video-assisted thoracic surgery; OT, open thoracotomy; LOS, length of stay.

## Discussion

With the advances in endoscopic equipment and surgical skills, VATS has become a useful modality for the management of many intrathoracic diseases. Compared with VATS esophagectomy and VATS dissection of mediastinal tumors, VATS lobectomy is increasingly being performed for early lung cancer worldwide. Hence, there is a tendency for VATS lobectomy to replace most open lobectomy procedures^15^.

In this VATS lobectomy series (NSCLC group), the postoperative morbidity and mortality rates were 2.4% and 0.4%, respectively. The postoperative LOS and chest tube duration were shorter in the VATS lobectomy group than in the OT lobectomy group, comparable with previous reports^2^^,^^3^. Swanson *et al*.^16^ also showed similar results. Hence, the safety of VATS lobectomy is comparable with that of OT lobectomy for operable NSCLC with reduced LOS and chest tube duration.

No significant difference was found in the number of LND and number of LND stations between VATS lobectomy and OT lobectomy for pathological stage I and II NSCLC. Watanabe *et al*.^17^ and Scott *et al*.^18^ demonstrated similar results. On the other hand, there was a significant difference in the number of LND stations for pathological stage III in the present study. Dellinger *et al*.^19^ found that the number of N_2_-level LND in the VATS lobectomy group is less than that in the OT group, and attributed the deficit in LND to the learning curve. Recently, Kim *et al*.^20^ found that for clinical stage I NSCLC patients with pathologic N_1_ or N_2_ disease after VATS lobectomy, survival was comparable with that after lobectomy through a thoracotomy. Therefore, based on literature and our experience, VATS has similar efficacy and results in similar long-term survival to OT for clinical early-stage NSCLC patients planned for lobectomy.

Despite considerable improvements in esophageal cancer staging and patient selection, generally high complication rates encourage the search for alternative operative techniques that can achieve similar cure rates but with less morbidity. With this objective, MIE has been introduced under the form of various combinations of thoracoscopy and laparoscopy. The VATS approaches for the treatment of esophageal cancer are currently still considered investigational and under clinical evaluation^4^^,^^5^^,^^21^^,^^22^. The indication for MIE is similar to that for OT, which is resectable esophageal cancer^4^.

This study showed no significant difference in the number of nodal dissection, chest tube duration, morbidity rate, mortality rate, and hospital LOS between the VATS esophagectomy and OE groups. However, the operation time was longer in the VATS esophagectomy group. Zingg *et al*.^21^ reported similar results. On the other hand, the morbidity rate after operation was still moderately high in our series; 7 patients had complications, including severe arrhythmias requiring medication in 3 cases, wound infection in 1 case, esophageal anastomotic leakages in 2 cases, and bronchial leakage in 1 case which resulted in the only death in our series. Luketich^12^ reported 27 anastomotic leaks accounting for 11.7% of the 222 patients, and tracheal tear was found in 2 patients. The overall mortality was 1.4% (3/222). Nguyen *et al*.^22^ reported 8 cases of gastrointestinal leaks and 4 cases of anastomotic intrathoracic leaks in 104 MIE operations, and the in-hospital mortality was 2.9%. Tan *et al*.^23^ reported 5 cases of anastomotic leaks in 36 MIE patients. However, no peri-operative death occurred because the esophagogastric anastomosis was in the neck. All these results showed that MIE can be safely performed only in large medical centers, although devastating results can occur after MIE.

Currently, there are two MIE techniques, including totally VATS esophagectomy and hybrid VATS esophagectomy. Although there is no evidence of a specific advantage of one technique over another^5^, minimally invasive Ivor-Lewis esophagectomy (thoracoscopy plus laparoscopy) with two-field lymph node dissection in patients with resectable esophageal cancer is the preferred approach in Pittsburgh Medical Center, the biggest MIE center for esophageal cancer in the USA^4^. With the development of surgical techniques, similar to total VATS pulmonary lobectomy becoming the gold standard for the surgical treatment of lung cancer requiring lobectomy^24^, total MIE may become the gold standard for the surgical treatment of resectable esophageal cancer in the near future.

Neurologic fibroma is safe for VATS dissection for benign mediastinal tumors, such as thymic cyst. The most common indication for VATS thymectomy is the treatment of myasthenia gravis^25^. The controversies surrounding the indication of VATS thymectomy for thymoma^6^^,^^7^^,^^26^^,^^27^ has two aspects, namely, tumor size and tumor staging. The median tumor size of thymoma was 5.4 cm in the VATS group, which was comparable with the 4.5 cm in the open thymectomy group in our series. Li  *et al*.^27^ reported that resections of noninvasive bulky solid thymomas by complete thoracoscopy are feasible and safe. The mean tumor size was 7.1 cm (5 to 10 cm) and patients survived 5 to 75 months without evidence of recurrence or metastasis. Pennathur *et al*.^7^ reported that 40 early-stage patients (14 of stage I and 26 of stage II) received thymectomy (open thymectomy for 22 patients and VATS for 18). No significant difference was found in the estimated recurrence-free and overall 5-year survival rates (83%-100%) between the two groups. The median tumor size of thymomas was 4 cm (3 to 8 cm), and all patients (4 of stage I and 7 of stage II) underwent complete dissection. Tumor size alone is not absolute contraindication. Therefore, based on literature and our results, VATS thymectomy for early-stage thymoma (Masaoka stage I and II) is safe and feasible.

In the present study, the VATS lobectomy patients were younger than those in the studies reported by Swanson *et al*.^16^ and Scott *et al*.^18^, in which the median ages were 66 and 72 years, respectively. On the other hand, the median age of 59 years in our lobectomy series was comparable with that in other medical centers in China. Yang *et al*.^28^ reported an average age of 59 years for patients receiving lobectomy in a multi-center series of 600 consecutive cases of VATS lobectomy. For thymoma patients, the median age of 52 years in our series was within the range (44 to 63 years) of previous reports^7^^,^^29^.

The operation time in the VATS group (including VATS esophagectomy and VATS thymectomy) was longer than that in the OT group. Learning curves are important in these procedures. Osugi *et al*.^30^ reached a plateau of VATS esophagectomy after performing 34 procedures. Toker *et al*.^31^ demonstrated that a thoracic surgeon must perform at least 30 VATS thymectomies to reach a success rate of 80%.

The median postoperative LOS of 9 days in the VATS lobectomy group in this study was longer than that in the studies of Swanson *et al*.^16^ and Scott *et al*.^18^, in which the median LOS values were 6.1 and 4.5 days, respectively. In the multi-center VATS lobectomy series of Yang *et al*.^28^ in China, the median LOS was 11.8 days, which was comparable to that in the present report.

This study had several limitations. First, it was retrospective. Second, only short-term outcomes were observed and long-term survival needs further evaluation. Finally, the results were obtained from one cancer center and may not be generalized to other medical centers.

## Conclusions

For early-stage (I/II) NSCLC patients who underwent lobectomy, VATS had similar short-term outcomes to those of OT approach. For pulmonary wedge resection, VATS was superior to the OT approach, with shorter chest tube duration and hospital LOS. For resectable esophageal cancer, VATS esophagectomy had similar morbidity and mortality to those of OT esophagectomy. There is a tendency for total MIE (thoracoscopy plus laparoscopy) to become the preferred approach for resectable esophageal cancer. VATS thymectomy for Masaoka stage I and II thymoma is feasible and safe, and tumor size is not contraindicated. Longer follow-ups are needed to determine the oncologic equivalency of VATS lobectomy, pulmonary wedge resection, esophagectomy, and thymectomy for thymoma versus OT.
